# Study of Post-COVID-19 Syndrome in Saudi Arabia

**DOI:** 10.7759/cureus.17787

**Published:** 2021-09-07

**Authors:** Manal H Mahmoud, Fahad A Alghamdi, Ghaida A Alghamdi, Loai A Alkhotani, Mohammad A Alrehaili, Dalia K El-Deeb

**Affiliations:** 1 Community Medicine, Ibn Sina National College for Medical Studies, Jeddah, SAU; 2 Community Medicine, Alexandria Faculty of Medicine, Alexandria, EGY; 3 Medicine, Ibn Sina National College for Medical Studies, Jeddah, SAU

**Keywords:** saudi arabia, covid-19, post covid-19 syndrome, pandemic, predisposing factors

## Abstract

Background

Post-COVID-19 syndrome may be predisposed by organ damage as a complication of COVID-19. Patients may experience persistent symptoms after recovering from their initial illness.

Objectives

To identify manifestations and predisposing factors for post-COVID-19 syndrome in Saudi Arabia.

Methods

A cross-sectional study was conducted from May 2021 through June 2021 using an online structured pre-coded closed-ended, pilot-tested questionnaire in Arabic. It included male and female inhabitants of Saudi Arabia aged 18 years and above with a past history of COVID-19 infection. Descriptive statistics were carried out for all variables. A univariate analysis Chi-square test and independent t-test were used. A p-value of less than 0.05 was considered significant.

Results

A total of 85.3% of post-COVID-19 cases had general manifestations: 77.3% had musculoskeletal and joint complaints, 61.3% had mental and psychological problems, 58.7% had gastrointestinal manifestations, 44% had renal complaints, 41.3% had respiratory complaints, and 36.0% had cardiovascular symptoms. Gender, age, smoking, BMI, associated morbid condition, number of previous COVID-19 attacks, the severity of illness, place of treatment, and complications of COVID-19 due to treatment or hospitalization were significantly correlated with the occurrence of post-COVID-19 syndrome.

Conclusion

Post-COVID-19 syndrome could be manifested by fatigue, malaise, myalgia, joint pain, depression, anxiety, sleep, memory and concentration disturbances, cough, dyspnea, palpations, arrhythmias, and chest pain. It may be influenced by male gender, smoking, old age, high BMI, comorbidities, and past COVID-19 attacks with regard to the number, place of treatment, and occurrence of complications.

## Introduction

COVID-19 is a zoonotic respiratory disease that started in Wuhan city in China and turned into a pandemic stated by World Health Organization (WHO) [1].

COVID 19 is manifested by cough, fever, shortness of breath, sudden onset of an abnormal smell (anosmia or cacosmia), loss, or impaired taste (ageusia or dysgeusia). Other manifestations include confusion, chills, fatigue, generalized myalgia, malaise, drowsiness, cough, acute respiratory distress syndrome with dyspnea and pneumonia, a severe gastrointestinal disorder with diarrhea, liver injury, coagulation dysfunction, cardiovascular failure, and other clinically relevant neurological involvement. It is confirmed by the detection of SARS-CoV-2 nucleic acid or antigen in a clinical specimen [2-4].

Although WHO planned recommendations to control the COVID-19 pandemic, waves of the pandemic are still present [5]. It has a mortality rate of 5-6%, with about 3.6 million worldwide deaths. It affected nearly 172 million people worldwide; 75% of them recovered either completely or with some complications. Arab countries of the Gulf, including the Kingdom of Saudi Arabia, are the least affected [6].

People who are in close contact with infected persons and those with impaired immunity and chronic medical conditions are more susceptible to the disease and its complications [1]. Mortality is higher among males in all age groups [7]. Complications of COVID-19 include acute respiratory failure, pneumonia, acute respiratory distress syndrome, acute liver injury, acute cardiac injury, secondary infection, acute kidney injury, septic shock as well as post-COVID-19 syndrome and death [8].

The post-COVID-19 syndrome is a syndrome of persistent symptoms or development of sequelae beyond 3 or 4 weeks from the onset of acute symptoms of COVID 19 attack regardless of the severity of this attack. It could be also defined as persistent symptoms and/or delayed or long-term complications of SARS-CoV-2 infection beyond 4 weeks from the onset of symptoms [9 -13]. Based on recent literature, it could be divided into two types: (1) subacute or ongoing symptomatic COVID-19, which includes symptoms and abnormalities present from 4-12 weeks beyond acute COVID-19; and (2) chronic or post-COVID-19 syndrome, which includes symptoms and abnormalities persisting or present beyond 12 weeks of the onset of acute COVID-19 and not attributable to alternative diagnoses [12].^ ^The main presentations are fatigue, shortness of breath or difficulty breathing, cough, joint, muscle, and chest pain, memory disturbances, concentration or sleep problems, headache, tachycardia, loss of smell or taste, depression or anxiety, fever, dizziness [9].

Post-COVID-19 syndrome may result in organ damage that resulted as sequelae of COVID-19, multisystem inflammatory syndrome following COVID 19 illness, blood clots, and leaking blood vessels that complicate COVID-19 may be another predisposing factor [9].

Little is known about the manifestations and predisposing factors for post-COVID-19 syndrome throughout the world and in Saudi Arabia. So this study was carried out to answer the following questions: What are the manifestations and predisposing factors of the post-COVID-19 syndrome in Saudi Arabia? 

## Materials and methods

Study context and design

A cross-sectional study using an online questionnaire conducted by researchers from Ibn Sina National College for Medical Studies (ISNC) starting from May 2021 through June 2021.

Sampling

A voluntary response convenient sample of male and female participants aged above 18 years old with a past history of COVID-19 infection were included. The sample size was arrived at using the margin of error approach as seen in the equation below:

n=Z2P(1−P)/d2

where n is the sample size, Z is the statistic corresponding to the level of confidence, P is expected prevalence (that can be obtained from the same studies or a pilot study conducted by the researchers), and d is precision (corresponding to effect size) [14].

The sample size was set at 150 participants divided into two groups according to whether they suffered from the post-COVID-19 syndrome or not.

n = Z2pq/ d2

Where,

Z2 = Z raised to power 2

d2 = d raised to power 2

n = minimum sample size

Z2 = Standard score corresponding to a given confidence level. Example, at 95% confidence level or 5% level of significance (a = 0.05), Z = 1.96.

P = Prevalence of disease is 1%

q = (1 - p) or percentage of failure which is 100 - 1 = 99%

d = Precision limit or proportion of sampling error, which is usually 5% confidence limit.

n = Z2P (1-q)/d2

The actual prevalence of COVID-19 in the Kingdom of Saudi Arabia was nearly 1.7 (about 600,000 cases with a total population of 35 million).

Here, in the equation, the prevalence used was .01 (as expected among the studied group, for response rate and duration and feasibility of the study)

Data collection methods

A structured pre-coded closed-ended, pilot-tested questionnaire in Arabic was developed. It included the following data: demographic data (age, sex, marital status, education, occupation, smoking history, and BMI), medical history, COVID-19 vaccination state, history of last COVID-19 attack, and clinical manifestation of post-COVID-19. The reliability of the questionnaire (content validity) was tested by SPSS Statistics (IBM Corp, Armonk, USA) using Cronbach’s alpha test (r = 0.89).

Statistical analysis

Data were collected and processed using SPSS version 22. Descriptive statistics were carried out for all variables. The participants were divided into two groups: Group I without post-COVID-19 syndrome and Group II with post-COVID-19 syndrome (according to the following: persistent symptoms of COVID-19 or development of sequelae beyond 4 weeks from the onset of acute symptoms of COVID-19 attack). A univariate analysis Chi-square test and independent t-test were used. A p-value < 0.05 was considered statistically significant. Spearman's correlation was used to study the predisposing factors for developing the post-COVID-19 syndrome.

Ethical considerations

Ethical approval for the study was obtained from Ibn Sina National College (ISNC) Research and Ethics Committee (IEC Ref No: H-04-03062021) in accordance with the Declaration of Helsinki for Human Studies [14]. Participation in the study was voluntary and during the online questionnaire, the participants were all informed about the purpose of the study and their right to refuse participation. Ethical conduct was maintained during data collection and throughout the research process.

## Results

The total number of participants that were included in the study was 150, divided into two groups. The first group was without post-COVID-19 syndrome and the second group suffered from post-COVID- 19 syndrome.

More than half (57.3%) of the participants were females, 54.7% were not married. The mean age of the study participants was 32.8 (5.52) years (p=0.042). Only 24.7% were nonsmokers, the remaining were either passive smokers (13.3%), cigarette smokers (44.7%), shisha smokers (10.0%), or vape smokers (7.3%) (p=0.039). The mean BMI was 25.9 (3.21) (p=0.041) (Table 1).

**Table 1:** Socio-demographic characters of the study participants *P-value is significant at less than 0.05 level Group I N=75 ( without post-COVID-19 syndrome) Group II N=75 ( with post-COVID-19 syndrome)

			Group I N=75 (%)	Group II N=75 (%)	Total N=150(%)	p-value
Gender	Male		33(44)	31(41.3)	64 (42.7)	0.041*
	Female		42(56)	44(58.7)	86 (57.3)	
Age in years Mean (Sd)			29.3 (6.91)	36.2 (7.65)	32.8 (5.52)	0.042*
Nationality	Non Saudi		12 (16.0)	14 (18.7)	26 (17.3)	0.501
	Saudi		63 (84.0)	61(81.3)	124 (82.7)	
Place of residence	Mekka Region		48 (64.0)	43 (57.3)	91 (60.7)	0.590
	Outside Mekka Region		27 (36.0)	32(42.7)	59 (39.3)	
Marital status	Never married		43 (57.3)	39 (52.0)	82 (54.7)	0.679
	Ever married		32 (427)	36 (48.00)	68 (45.3)	
Education	Basic education		6 (8.0)	8 (10.7)	14 (9.3)	0.725
	Secondary school		20(26.7)	16 (21.3)	36 (24.0)	
	University and more		49 (65.3)	51(68.0)	100(66.7)	
Work status	Not working		32 (42.7)	27(36.0)	59 (39.3)	0.810
	Private sector		13 (17.3)	11 (14.7)	24 (16.0)	
	Governmental sector		30 (40.0)	37 (49.3)	67 (44.7)	
Smoking history	Non smoker		23 (30.7)	14 (18.7)	37 (24.7)	0.039*
	Passive smoker		12(16.0)	8 (10.7)	20 (13.3)	
	Active Smoker	Cigarette	29 (38.7)	38 (50.7)	67 (44.7)	
		Shisha	10 (13.3)	5 (6.7)	15 (10.0)	
		Vape	6 (8.0)	5 (6.7)	11(7.3)	
BMI Mean (Sd)			22.4 (5.66)	29.6 (4.78)	25.9 (3.21)	0.041*

Group II significantly had 45.3% with cardiovascular diseases, 57.3% with respiratory diseases, and 27.3% with hepatic diseases (p=0.042, 0.032, 0.046 respectively). Nearly half of the participants (48.0%) were partially vaccinated (received one dose) and 29.3% were fully vaccinated (Table 2).

**Table 2:** Associated morbid conditions of the study participants *P-value is significant at less than 0.05 level Group I N=75 ( without post-COVID-19 syndrome) Group II N=75 ( with post-COVID-19 syndrome)

			Group I N=75 (%)	Group II N=75 (%)	Total N=150(%)	p-value
Cardiovascular	No		62 (82.7)	41(54.7)	103 (68.7)	0.042*
	Yes		13 (17.3)	34 (45.3)	47 (31.3)	
Respiratory	No		47 (62.7)	32(42.7)	79 (52.7)	0.032*
	Yes		28 (37.3)	43 (57.3)	71 (47.3)	
Endocrine	No		66 (88.0)	54 (72.0)	120 (80.0)	0.610
	Yes		9 (12.0)	21 (28.0)	30 (20.0)	
Liver	No		63 (84.0)	46 (61.3)	109 (72.7)	0.046*
	Yes		12 (16.0)	29 (38.7)	41(27.3)	
Renal	No		51(68.0)	48 (64.0)	99 (66.0)	0.052
	Yes		24(32.0)	27 (36.0)	51(34.0)	
Vaccination	No		13(17.3)	21 (28.0)	34 (22.7)	0.134
	Yes	Partial	39 (52.0)	33(44.0)	72 (48.0)	
		Full	23 (30.7)	21(28.0)	44 (29.3)	

Table 3 illustrates that dyspnea, fever, cacosmia, loss of taste and headache were the dominant symptoms of the COVID-19 attack of the studied participants (85.3%, 82.7%, 82.7%, 67.3%, 57.3%, respectively; p=0.033, 0.142, 0.029, 0.036 and 0.061, respectively). Nearly half (48.7%) of past COVID-19 cases were treated at home (p=0.046), 50.7% were mild cases (p=0.039), and 60.7% completely cured immediately after infection (p=0.035) . Complications that occurred due to treatment or hospitalization for last COVID-19 attack included fatigue (63.3%; p=0.029), post-traumatic stress disorder (54.0%; p=0.461), and malaise (39.3%; p=0.032) (Table 3).

**Table 3:** Description of previous COVID-19 attack of the studied participants *P-value is significant at less than 0.05 level Group I N=75 ( without post-COVID-19 syndrome) Group II N=75 ( with post-COVID-19 syndrome)

		Group 1 N=75 (%)	Group II N=75 (%)	Total N=150(%)	p-value
Number of attacks: Mean (Sd)		1.3 (1.26)	1.9(1.45)	1.7 (0.95)	0.065
Symptoms present when infected					
Fever	No	15 (20.0)	11 (14.7)	26 (17.3)	0.142
	Yes	60 (80.0)	64 (85.3)	124 (82.7)	
Dyspnea	No	13 (17.3)	9 (12.0)	22 (14.7)	0.033*
	Yes	62 (82.7)	66 (88.0)	128 (85.3)	
Cacosmia	No	18 (24.0)	8 (10.7)	26 (17.3)	0.029*
	Yes	57 (76.0)	67(89.3)	124 (82.7)	
Loss of taste	No	26 (34.7)	23 (30.7)	49 (32.7)	0.036*
	Yes	49 (65.3)	54 (69.3)	101 (67.3)	
Headache	No	33 (44.0)	31(41.3)	64 (42.7)	0.061
	Yes	42 (56.0)	44 (58.7)	86 (57.3)	
Diarrhea	No	38 (50.7)	36 (48.0)	74 (49.3)	0.561
	Yes	37 (49.3)	39 (52.0)	76(50.7)	
Constipation	No	45 (60.0)	47 (62.7)	92 (61.3)	0.438
	Yes	30 (40.0)	28 (37.3)	58(38.7)	
Place of treatment					
Home		43 (53.3)	30 (40.0)	73(48.7)	0.046*
Hospital		20 (26.7)	16 (21.3)	36 (24.0)	
ICU		12 (16.0)	29 (38.7)	41(27.3)	
Severity of illness					
Mild		41(54.7)	35 (46.7)	76 (50.7)	0.039*
Moderate		23 (30.7)	31 (41.3)	54(36.0)	
Sever		11 (14.6)	9 (12.0)	20(13.3)	
Complete cure after infection	No	14 (18.7)	45 (60.0)	59 (39.3)	0.035*
	Yes	61 (81.3)	30(40.0)	91 (60.7)	
COVID-19 Complications due to treatment or hospital admission					
Malaise	No	52 (69.3)	39 (52.0)	91 (60.7)	0.032*
	Yes	23 (30.7)	36 (48.0)	59 (39.3)	
Fatigue	No	34 (45.3)	21 (28.0)	55 (36.7)	0.029*
	Yes	41(54.7)	56 (72.0)	95 (63.3)	
Post-traumatic stress disorder	No	33 (44.0)	36 (48.0)	69 (46.0)	0.461
	Yes	42 (56.0)	39 (52.0)	81 (54.0)	
Post-intensive care syndrome	No	61(81.3)	52 (69.3)	113 (75.3)	0.049*
	Yes	14 (18.7)	23 (30.7)	37 (24.7)	

**Figure 1:**
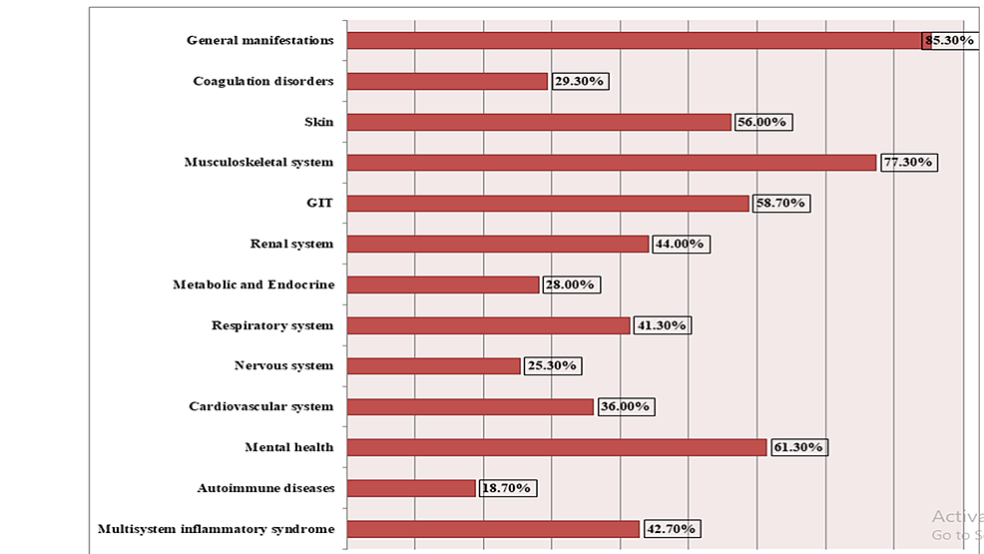
Clinical presentations of Group II studied participants (post-COVID-19) GIT: gastrointestinal tract

The majority of those who had post-COVID-19 syndrome (85.3%) had general manifestations (as malaise, fatigue, headache, and dizziness), 77.3% had musculoskeletal and joint complaints, 61.3% had mental and psychological problems (as depression, anxiety, memory, concentration and sleep disturbances, smell or taste disturbances), 58.7% had gastrointestinal manifestations (as dyspepsia), 44% had renal complaints, 41.3% had respiratory complaints (as dyspnea and cough), and 36.0% had cardiovascular symptoms (as chest pain, palpitations, and tachycardia) (Figure 1).

Gender, age, smoking, BMI, associated co-morbid condition, number of previous COVID-19 attacks, the severity of illness, place of treatment, and complications of COVID-19 due to treatment or hospitalization were significantly correlated with the occurrence of the post-COVID-19 syndrome (Table 4). 

**Table 4:** Significant correlations with post-COVID-19 syndrome *Correlation is significant at 0.05 (two-tailed) **Correlation is significant at 0.01 (two-tailed)

	r	Sig.
Gender	-.87	0.000**
Age	.73	0.030*
Smoking history	.79	0.001**
BMI	.68	0.002**
Associated morbid conditions	.59	0.014*
Number of attacks of previous COVID-19 infection	.47	0.023*
Severity of illness	.51	0.000**
Place of treatment	.63	0.012*
COVID-19 Complications due to treatment or hospital admission	.58	0.033*

## Discussion

This research was designed to identify manifestations and predisposing factors for post-COVID-19 syndrome in Saudi Arabia. The participants were divided according to the presence of post-COVID-19 manifestations into two equal groups; each included 75 participants namely Group I without post-COVID-19 manifestations and Group II with post-COVID-19 manifestations.

The post-COVID-19 syndrome is a syndrome of persistent symptoms or development of sequelae beyond 3 or 4 weeks from the onset of acute symptoms of COVID-19 attack regardless of the severity of this attack [9-12]. The exact mechanism for the syndrome, in general, is still unknown. Yet, one recent study [9] Which investigated the mechanism of severe post-COVID-19 syndrome form of the multisystem inflammatory syndrome in children determined that viral particles remaining in the gut long after initial COVID-19 infection and could pass into the bloodstream [9]. Another research [15] claimed that the mechanism of its development may be direct viral toxicity; endothelial damage and microvascular injury; immune system impaired regulation and stimulation of a hyperinflammatory state; hypercoagulability with resultant in situ thrombosis and macrothrombosis; and maladaptation of the angiotensin-converting enzyme 2 (ACE2) pathway [15].

The current work concomitant with other researches demonstrated that fever, dyspnea, anosmia/ cacosmia, and loss of taste were the most frequently encountered clinical manifestations of COVID-19. [9,16-19] The severity of these symptoms varies according to the presence of underlying morbid conditions [16, 20]. The present work revealed that respiratory, hepatic, renal, and cardiovascular comorbidities were encountered among the great proportion of the studied participants; especially those having post-COVID-19 syndrome. Moreover, these co-morbid conditions may predispose to the development of post-COVID-19 syndrome [15,21]. 

The present work revealed that the main manifestations encountered among those with the post-COVID-19 syndrome were general manifestations (as fatigue, and malaise), musculoskeletal (muscle and joint pain), mental health (depression, anxiety, sleep, memory and concentration disturbances), respiratory (cough and dyspnea) and cardiovascular (palpations, arrhythmias, and chest pain). The same findings were reported by a comprehensive review of current literature on post-COVID-19 syndrome done by Nalbandian A et al on April 2021 [22].

Mental stress, in general, has been reported to impair immunity, therefore, increasing susceptibility for diseases [23]. Moreover, infection with COVID-19 will negatively affect a patient’s life, sleep pattern, and mood. That will predispose him again to a vicious circle of depression, stress and anxiety, and impaired immunity [24-26]. These findings were in coherence with our study results which revealed that a great portion of the study participants with post-COVID-19 syndrome experienced headaches, mental complaints in form of anxiety, depression, and sleep disturbances during their last COVID-19 attack. Those symptoms would precipitate stressful conditions that lower immunity and therefore predisposed them to post-COVID-19 syndrome [23-16].

Until now, there is no promising treatment option for COVID-19, and the drugs used have many toxic side effects [27]. This may explain our result of a high percentage of reported complications among post-COVID-19 syndrome patients treated from their last COVID-19 attack at hospitals. Another explanation is that those cases were severe enough to develop complications on their own so that they need hospitalization or intensive care admission.

The present study demonstrates that almost all study participants without post-COVID-19 syndrome had more vaccination coverage whether partially (with only one dose) or fully vaccinated (with two doses). Effective vaccines are important to prevent and control epidemics of emerging viruses as SARS-CoV-2 [28,29]. ^ ^Vaccination prevents severe forms and hospitalization due to COVID-19 infection including post-COVID-19 syndrome.

The present study proved that male gender, smoking, increase in age, high BMI, associated comorbidities, number, place of treatment, severity, and associated complications of COVID-19 attacks; all were correlated with the development of the post-COVID-19 syndrome. Nalbandian A et al discussed all those parameters and considered them also as predisposing factors for the development of post-COVID-19 syndrome [22].

## Conclusions

The post-COVID-19 syndrome is a serious complication following COVID-19, where some manifestations persist or developed after 4 weeks of COVID-19 attack and after a negative polymerase chain reaction (PCR) test. In Saudi Arabia, as in other countries, it was found that it could be manifested by fatigue, malaise, myalgia, joint pain, depression, anxiety, sleep, memory and concentration disturbances, cough, dyspnea, palpations, arrhythmias, and chest pain. It may be predisposed by male gender, smoking, old age, high BMI, comorbidities, and past COVID-19 attack with regard to the number, place of treatment, and occurrence of complications.
